# Comparative Assessment of Antitumor Effects and Autophagy Induction as a Resistance Mechanism by Cytotoxics and EZH2 Inhibition in INI1-Negative Epithelioid Sarcoma Patient-Derived Xenograft

**DOI:** 10.3390/cancers11071015

**Published:** 2019-07-19

**Authors:** Silvia Stacchiotti, Valentina Zuco, Monica Tortoreto, Denis Cominetti, Anna Maria Frezza, Stefano Percio, Valentina Indio, Marta Barisella, Valentina Monti, Silvia Brich, Annalisa Astolfi, Chiara Colombo, Sandro Pasquali, Marco Folini, Mrinal M. Gounder, Maria A. Pantaleo, Paola Collini, Angelo Paolo Dei Tos, Paolo Giovanni Casali, Alessandro Gronchi, Nadia Zaffaroni

**Affiliations:** 1 Adult Mesenchymal Tumor and Rare Cancer Unit, Department of Cancer Medicine, Fondazione IRCCS Istituto Nazionale Tumori, via Venezian 1, 20133 Milan, Italy; 2 Molecular Pharmacology Unit, Department of Applied Research and Technological Development, Fondazione IRCCS Istituto Nazionale Tumori, Via Amadeo 42, 20133 Milan, Italy; 3 Department of Biomedical and Clinical Sciences L. Sacco, University of Milan, Via Grassi 74, 20157 Milan, Italy; 4 Interdepartmental Centre of Cancer Research “Giorgio Prodi”, University of Bologna, Via Zambini 33, 40126 Bologna, Italy; 5 Department of Pathology, Fondazione IRCCS Istituto Nazionale Tumori, via Venezian 1, 20133 Milan, Italy; 6 Department of Surgery, Fondazione IRCCS Istituto Nazionale Tumori, via Venezian 1, 20133 Milan, Italy; 7 Sarcoma Medical Oncology and Early Drug Development, Memorial Sloan Kettering Cancer Center, 1275 York Avenue, New York, NY 10065, USA; 8 Department of Medicine, University of Padua School of Medicine, Via Giustiniani 2, 35128 Padua, Italy

**Keywords:** PDX, epithelioid sarcoma, EZH2, autophagy, HMGA2

## Abstract

Epithelioid sarcoma (ES) is a rare mesenchymal malignancy marked by SMARCB1/INI1 deficiency. Retrospective clinical data report on the activity of anthracycline- and gemcitabine-based regimens. EZH2 inhibitors are currently being tested in clinical trials. Since comparisons of these agents are unlikely to be prospectively evaluated in the clinics, we took advantage of an INI1-deficient proximal-type ES patient-derived xenograft (PDX ES-1) to comparatively assess its preclinical antitumor activity. Mice were treated with doxorubicin and ifosfamide, singly or in combination, gemcitabine, and the EZH2 inhibitor EPZ-011989. Comparable antitumor activity (max tumor volume inhibition: ~90%) was caused by gemcitabine, EPZ-011989, and the doxorubicin–ifosfamide combination. The integration of RNAseq data, generated on tumors obtained from untreated and EPZ-011989-treated mice, and results from functional studies, carried out on the PDX-derived ES-1 cell line, revealed autophagy induction as a possible survival mechanism in residual tumor cells following EPZ-011989 treatment and identified HMGA2 as a main player in this process. Our data support the clinical use of gemcitabine and the doxorubicin–ifosfamide combination, confirm EZH2 as a therapeutic target in proximal-type ES, and suggest autophagy as a cytoprotective mechanism against EZH2 inhibition.

## 1. Introduction

Epithelioid sarcoma (ES) is a very rare subtype of soft tissue sarcoma (STS), most often occurring in adolescents and young adults, which has distinctive clinical and histopathological features [1,2]. Patients affected by ES exhibit a propensity for local recurrence as well as for metastatic spread to loco-regional lymph nodes and the lungs. Two subtypes of ES harboring different histological features are currently recognized: (i) Distal-type ES (D-ES), occurring most frequently, but not exclusively, in the distal extremities and (ii) proximal-type ES (P-ES), more frequently arising in proximal sites [2,3,4]. Patients affected by D-ES and P-ES have different outcomes, with P-ES being characterized by a more aggressive tumor biology [5,6]. Surgery with or without radiotherapy is the standard treatment option for both localized D-ES and P-ES, while systemic chemotherapy is used for advanced cases [5,6].

Only retrospective data are available so far on the activity of cytotoxic chemotherapy in ES [7,8,9,10,11]. In the largest series published on INI1-negative ES patients suffering advanced disease, which was derived from a multicentric, international retrospective study aimed at investigating the activity of the medical agents formally approved for treatment of this disease, drug efficacy looked to be limited, especially in terms of the duration of response. In details, the RECIST overall response rate (ORR) was 22% of 85 patients treated with anthracycline-based chemotherapy, which represents the front-line standard treatment for advanced STS, for a six-month median progression-free survival (PFS). A trend toward a higher response rate was observed in P-ES (26%) versus D-ES (19%) [12].

In the same study, gemcitabine-based regimens administered in 41 patients achieved a 27% RECIST ORR, with a four-month median PFS and a trend toward a higher response rate in the distal than in the proximal morphological type (30% versus 22%) [12]. No RECIST responses were detected in ES patients treated with pazopanib (median PFS of three months) [12]. These results confirmed that new active drugs are strongly needed in the front-line treatment of this disease.

Recent studies demonstrated that alterations of the chromatin setting significantly influence tumorigenesis of ES. Specifically, ES is almost invariably marked by deficiency of INI1, a component of the SWI/SNF chromatin-remodeling complex encoded by the oncosuppressor gene *SMARCB1* [13], which is involved in active gene transcription [14,15]. Loss of nuclear expression of the INI1 protein (INI1 deficiency) is characteristic of ES, can be detected by immunohistochemistry (IHC), and is used for diagnostic purposes [14]. INI1 loss impairs SWI/SNF function, leading to unimpeded oncogenic activity of Enhancer of Zeste Homolog 2 (EZH2), a histone methyltransferase and the core enzymatic subunit of the polycomb repressive complex 2 (PRC2). EZH2 catalyzes tri-methylation of histone H3 at Lys 27 (H3K27me3) to negatively regulate gene expression and acts as an oncogenic driver in tumor cells [16]. Immunohistochemical deficiency of INI1 can be variably due to gene deletion, gene mutation, or epigenetic modifications [17,18].

Pharmacologic inhibition of EZH2 was demonstrated to reduce tumor growth and promote apoptosis in preclinical models of different tumor types characterized by the presence of EZH2-activating mutations [19,20] or EZH2 altered expression [21,22]. These data are now supported by a recently published Phase I clinical study, where the clinical response of the orally available drug EPZ-6438 (Tazemetostat) was assessed in relapsed/refractory B-cell non-Hodgkin lymphoma and advanced solid tumors. Particularly, a clinical benefit was achieved in 5 of 13 patients with IN1-negative or SMARCA4-negative tumors (38%), including two patients with ES who had prolonged stable disease for more than 20 months [23]. These results were the basis for a Phase 2 prospective international multicentric basket clinical study investigating the activity of tazemetostat in several different solid tumor types, among which advanced INI1-negative ES was a category. In the ES cohort, 62 patients received tazemetostat, which showed an ORR by RECIST of 15%, with 56% stable disease and 21% progression as the best response. Interestingly, 51% of patients experienced tumor shrinkage. The median PFS was 23 weeks, with 21% of patients progression-free at 1 year [24].

Given the rarity of ES, a formal comparison of different available agents showing activity in the disease is unlikely to be prospectively evaluated in clinical trials. We took advantage of the availability of a recently established patient-derived xenograft (PDX), obtained by a human, INI-deficient, naïve, primary P-ES, which was directly xenotransplanted into severe combined immunodeficient (SCID) mice to comparatively assess the activity of doxorubicin and ifosfamide, as single agents and in combination, gemcitabine, and the small molecule EZH2 inhibitor EPZ-011989 [25]. Results are reported herein.

## 2. Results

### 2.1. Experimental Model Characterization

**Figure 1:**
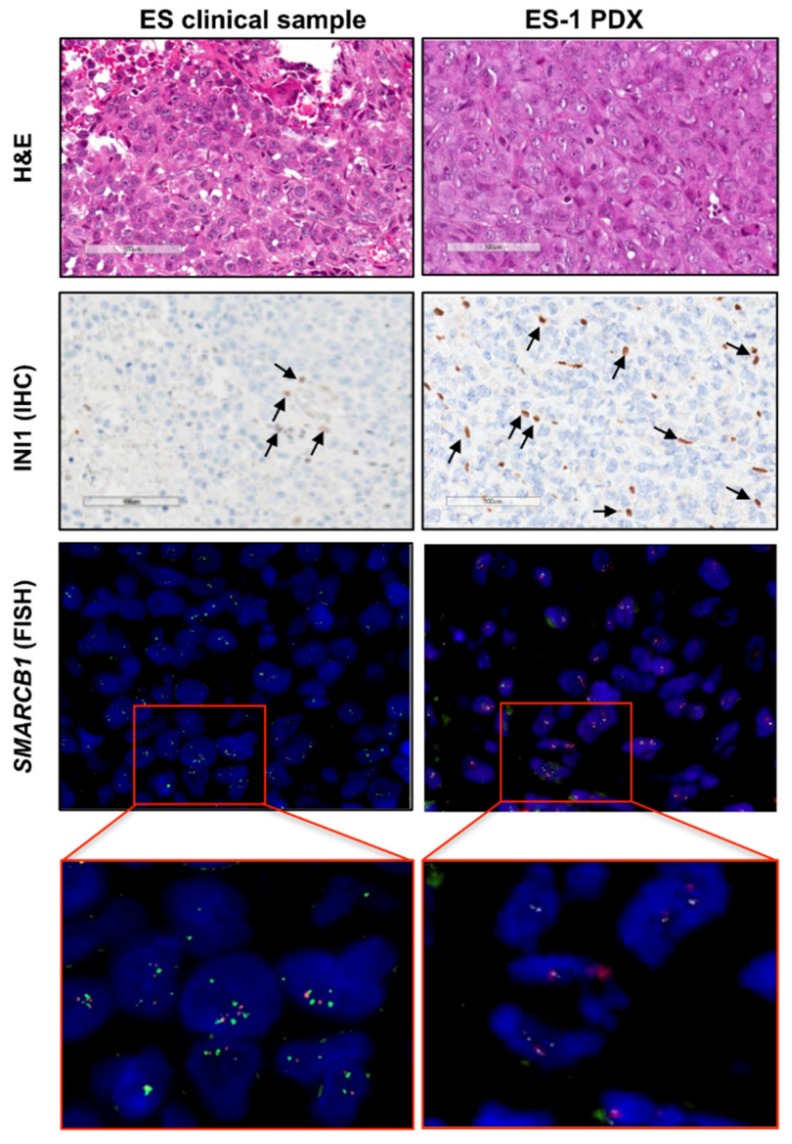
Characterization of the patient-derived xenograft (PDX) model in comparison to the original clinical tumor. Representative pictures of the PDX model (epithelioid sarcoma (ES)-1 PDX) and corresponding clinical tumor (ES clinical sample) are shown. The histology was assessed on hematoxylin and eosin (H and E)-stained slides (upper panels). INI1 deficiency was detected at the protein level by INI1 immunostaining (INI1) (intermediate panels) (scale bar = 100 μm). INI1-positive intratumoral lymphocytes (internal positive control) are indicated by arrows. FISH analysis by a specific SPEC SMARCB1/22q12 Dual Color probe showed no *SMARCB1* gene deletion (lower panels). The green fluorochrome-labeled probe hybridizes the human *SMARCB1* gene on the chromosomal region 22q11.23; the orange fluorochrome-labeled probe hybridizes the *KREMEN1* gene region in 22q12.1-q12.2. Magnification, 100×.

The patient-derived tumor sample was consistent with a P-ES, composed by epithelioid and even rhabdoid cells, with large cytoplasms and nuclei with prominent nucleoli, growing as sheets with little or no stroma interposed and scarce lymphocytic infiltrate [2]. The histomorphology of the ES-1 PDX model was superimposable to that of the clinical tumor (Figure 1, upper panels). In addition, both samples showed an absence of INI1 nuclear immunohistochemical staining in tumor cells (Figure 1, intermediate panels). In both tumor samples, no deletion of *SMARCB1* gene was detected by FISH (Figure 1, lower panels), indicating that the loss of the INI1 protein was due to either *SMARCB1* gene mutation or epigenetic modification.

### 2.2. Pharmacological Studies

#### 2.2.1. Antitumor Activity Studies

**Figure 2:**
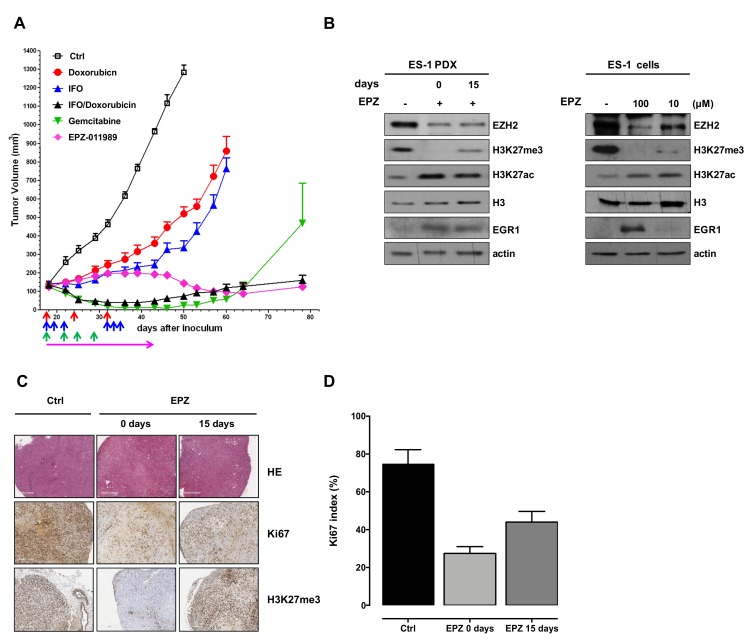
Antitumor activity and biochemical effects induced by EPZ-011989 in ES-1 PDX and derived cell line. (**A**) Antitumor activity of doxorubicin, ifosfamide (IFO), singly administered and in combination, gemcitabine, and EPZ-011989 (EPZ) in ES-1 PDX. Eight mice per experimental group were used. Growth curves report the average tumor volume (±SE) in control and drug-treated animal groups. The arrows in the figure indicate when the drugs were administered. (**B**) Western blot analysis of trimethylated and acetylated lysine 27 of histone H3 (H3K27me3 and H3K27ac, respectively), total histone H3, EZH2, and EGR1 levels in ES-1 tumors removed from untreated (−) and EPZ-011989-treated mice at different intervals from the end of treatment (left panel) and in ES-1 untreated and EPZ-011989-treated cells with different concentrations of EPZ-011989 for 96 h (right panel). Cropped images of selected proteins are shown. A representative Western blot of three independent experiments is shown. (**C**) Pathologic evaluation of ES-1 tumors obtained from untreated (Ctrl) and EPZ-011989-treated mice at different intervals from the end of treatment. Ki67 and H3K27me3 immunostaining of the same tumors are also presented. (scale bars = 500 µm for HE, 400 µm for Ki67 and H3K27me3). (**D**) Quantification of Ki67 index in untreated and EPZ-011989-treated tumors. Data are reported as mean ± SD of six different fields.

As single agents, both doxorubicin and ifosfamide showed a modest antitumor effect, with a maximum tumor volume inhibition (max TVI) of 62% and 75%, respectively. Notably, drugs were delivered at suboptimal doses to highlight the effects of the combined treatment. Indeed, the doxorubicin–ifosfamide combination showed enhanced antitumor activity compared with single agents, inducing an almost complete tumor growth inhibition (max TVI: 94%). Comparable antitumor activity was caused by gemcitabine (max TVI: 98%), although, 30 days after the end of treatment, tumors started to re-grow. EPZ-011989 initially induced a slight tumor growth delay compared to controls which was not sufficient to reach a complete tumor stabilization, but was then followed by a progressive reduction of tumor volume starting from the end of the treatment and still appreciable at the end of the experiment. A max TVI of 89% was induced by the drug, which was, however, significantly lower (*p* < 0.05) than that caused by the doxorubin–ifosfamide combination or gemcitabine (Figure 2A, Table 1). No sign of toxicity, in terms of tumor weight loss and occurrence of toxic death, was registered with any tested drug.

Consistent with its mechanism of action, EPZ-011989 caused a marked inhibition of H3K27 trimethylation (H3K27me3) levels, resulting in an epigenetic shift to H3K27 acetylation (H3K27ac), as detected by Western blotting in ex vivo tumor samples obtained at different intervals from the end of mouse treatment (Figure 2B). Interestingly, inhibition of EZH2 activity by EPZ-011989 also induced EZH2 protein down-regulation concomitantly with an increased expression of the known EZH2 target gene early growth response 1 (*EGR1*), a multifunctional transcription factor thought to act as a cancer suppressor [28] (Figure 2B). Consistent results were observed in the ES-1 cell line following 96 h of exposure to different EPZ-011989 concentrations, 10 and 100 µM, which did not appreciably affect cell growth, in agreement with previously reported findings on a B-cell lymphoma cell line [25].

#### 2.2.2. Histopathologic Evaluation of EPZ-011989-Treated ES Xenografts

In spite of the reduction in tumor volume, EPZ-011989-treated tumor samples excised from mice immediately or 15 days after the end of treatment showed an amount of viable tumor cells superimposable to that observed in untreated controls, although the proliferation rate, as detected by Ki67 index, was lower in treated compared to untreated samples (Figure 2C,D). With respect to untreated tumors, hematoxylin and eosin (H and E) staining of treated tumors showed only focal spindling of the tumor cells with loss of prominent nucleoli and dispersed chromatin, but no necrotic/apoptotic areas (Figure 2C) and no evidence of post-treatment changes (i.e., sclerojalinosis, histiocytic reaction with hemosiderophages, necrosis). Moreover, consistent with the Western blot results (Figure 2B), we found an almost complete loss of H3K27me3 immunohistochemical staining in tumors obtained from mice immediately at the end of treatment with EPZ-011989 (Figure 2C).

#### 2.2.3. EPZ-011989-Induced Gene Expression Modulation in ES Xenografts

Based on the evidence that, in spite of a significant tumor growth delay induced by EPZ-011989, tumor samples excised from treated mice were characterized by the presence of a high amount of viable tumor cells, although with a lower proliferative potential, we hypothesized that ES cells could activate fail-safe mechanisms to counteract the stress induced by EPZ-011989. In this respect, we investigated whether the drug modulated the expression of genes involved in cytoprotective mechanisms by RNA sequencing on tumors obtained from two control (untreated) and two EPZ-011989-treated mice.

**Figure 3:**
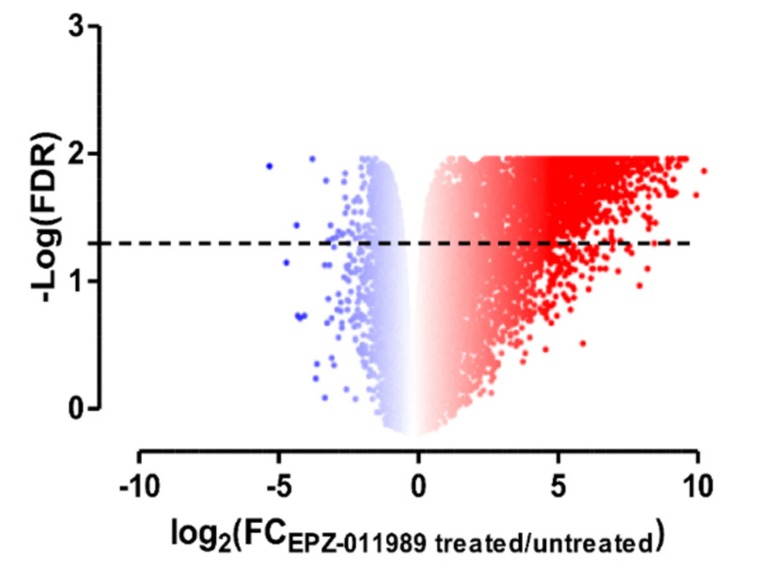
EZH2 inhibition leads to significant gene expression changes in ES-1 PDX. Volcano plot of genes differentially expressed between untreated and EPZ-011989-treated tumor samples; the red–blue colormap is used as a graphical visualization of the magnitude of log2 fold change (FC) representing the positive and negative values, respectively.

Differential gene expression analysis of tumor samples removed from EPZ-011989-treated and untreated mice revealed that, among the 5521 genes with a significant differential expression (according to false discovery rate, FDR < 0.05), 3410 genes (62%) were up-regulated and 2111 genes (38%) were down-regulated, respectively (Figure 3, Table S3), thus indicating a generally increased transcriptional activity in treated tumors, consistent with EZH2 inhibition. In line with this finding, a Gene Set Enrichment Analysis (GSEA) run using “EZH2” as a query in MSigDB database revealed 12 gene sets significantly enriched (FDR < 0.05) in the up-regulated genes upon EZH2 inhibition (Table S1). Among them, five deal with epigenetic modifications and transcriptionally repressed EZH2 targets (highlighted in Table S1).

Interestingly, among the top 15 up-regulated genes in the EPZ-011989-treated samples, eight are involved in cell survival pathways (Table 2), including insulin-like growth factor-binding protein 4 (*IGFBP4*) which promotes senescence in mesenchymal cells [29]; decorin (*DCN*) [30], high mobility group A2 (*HMGA2*) [31], secreted protein, acidic, and rich in cysteine (*SPARC*) [32], and aldehyde dehydrogenase 1A1 (*ALDH1A1* [33]), which are involved in autophagy induction; and EGF-containing fibulin-like extracellular matrix protein 1 (*EFEMP1*) [34], inositol polyphosphate 4-phosphatase type II (*INPP4B*) [35], and near insulin-induced gene 2 (*INSIG2*) [36], which counteract apoptosis in different tumor contexts.

Based on the notion that EZH2 is involved in the epigenetic control of autophagy [37], we further explored the potential involvement of autophagy in tumor response to EPZ-011989. GSEA analysis was carried out using three gene sets labeled as “autophagy” from the MSigDB database and a custom signature made of genes known to be related to autophagy machinery/regulation [38,39] (Table S2). Results showed that these gene sets were mostly affected by EPZ-011989 treatment. Although not statistically significant, possibly due to the scanty number of samples analyzed, the gene distribution confirmed an enrichment trend in the up-regulated genes upon EPZ-011989 exposure (Figure S1).

#### 2.2.4. Induction of Autophagy Has a Cytoprotective Role in EPZ-011989-Treated PDX and Cell Models

**Figure 4:**
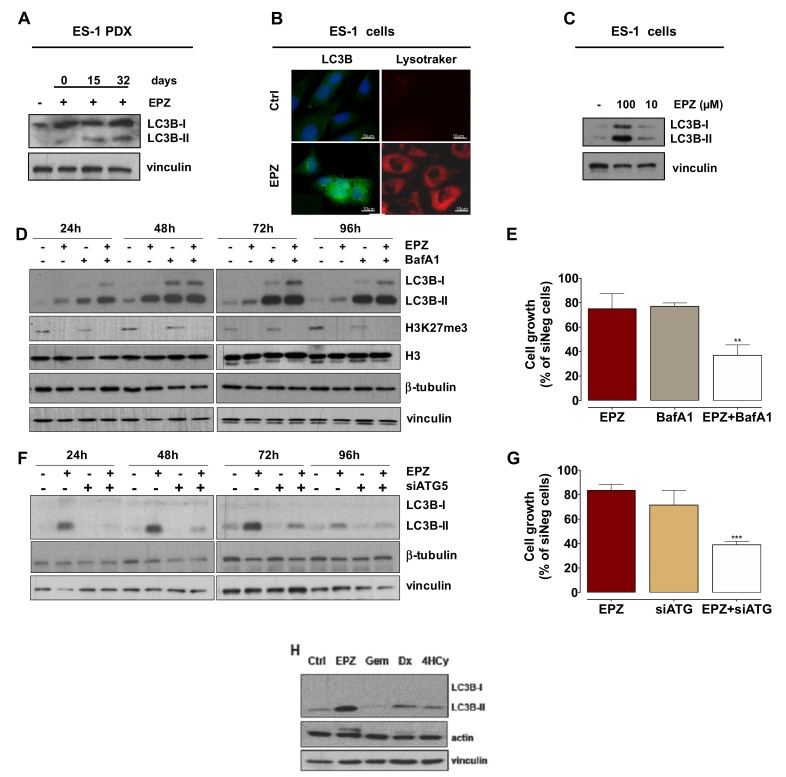
EPZ-011989 induces autophagy in ES-1 PDX and derived cell line. (**A**) Western blot analysis of LC3B in tumors obtained from untreated and EPZ-011989-treated mice at different intervals from the end of treatment. A representative Western blot of three independent experiments is shown. (**B**) Fluorescence microscopy images of untreated (control) or EPZ-011989-treated (100 µM for 96 h) ES-1 cells stained for LC3B (green) and LysoTrackerTM (red). Nuclei were counterstained with Hoechst 33342. Scale bar, 10 μm. Data are representative of three independent experiments. (**C**) Western blots showing LC3B conversion in untreated (control) or EPZ-011989-treated (10 and 100 µM for 96 h, EPZ) ES-1 cells. A representative Western blot of three independent experiments is shown. (**D**) Immunoblotting showing the time-course analysis of LC3B turnover and H3K27me3 and total histone H3 expression in untreated (−), EPZ-011989 (EPZ, 100 µM)-, BafilomicinA1- (BafA1, 30 nM) and EPZ+BafA1-treated ES-1 cells. A representative Western blot of three independent experiments is shown. (**E**) ES-1 cell growth inhibition after exposure to 100 µM EPZ-011989, 30 µM BafA1, or the combination of both for 96 h. Data are reported as percentage of treated cells (mean ± SD of three independent experiments) compared with untreated cells. ** *p* < 0.01. (**F**) Immunoblotting showing the time-course analysis of LC3B turnover in untreated (−) and EPZ-011989 (EPZ, 100 µM)-treated ES-1 cells after transfection with siATG5 (+) or siNeg (−). A representative Western blot of three independent experiments is shown. (**G**) Cell growth inhibition after 96 h exposure to 100 µM EPZ-011989 of ES-1 cells transfected with siATG5 or siNeg. Data are reported as a percentage of treated cells (mean ± SD of three independent experiments) compared with untreated siNeg cells. *** *p* < 0.005. (**H**) Western blot analysis of LC3B in ES-1 cells exposed for 72 h to different drugs: EPZ-011989 (EPZ, 100 µM), gemcitabine (Gem, 10 µM), doxorubicin (Dx, 0.1 µM), and 4-hydroperoxycyclophosphamide (4HCy, 1 µM). A representative Western blot of three independent experiments is shown. Cropped images of selected proteins are shown in all different Western blots.

Since transcriptome analysis revealed a positive enrichment of genes belonging to autophagy-related pathways, we assessed the conversion of the autophagy-associated marker LC3B, from the cytosolic LC3B-I form to the phosphatidylethanolamine-conjugated LC3B-II form, which is associated with autophagosomes [38]. Tumor samples obtained from EPZ-011989-treated mice at different intervals from the end of treatment consistently showed LC3B-II accumulation, thus indicating the occurrence of autophagy in residual tumor cells after treatment (Figure 4A). This observation gained further support by the evidence that, upon a 96-h exposure to EPZ-011989 ES-1, cells grown in vitro were characterized by a marked accumulation of the autophagosome marker, as well as by a pronounced enhancement of acid organelle labeling (Figure 4B). Altogether, these findings suggest the induction of autophagy in response to EZH2 inhibition, as also supported by the marked and dose-dependent conversion of LC3B-I to LC3B–II in EPZ-011989-treated cells (Figure 4C).

To validate the hypothesis that autophagy could represent a cytoprotective mechanism consequent to EZH2 inhibition, we characterized the autophagic flux in ES-1 cells exposed to EPZ-011989 through the analysis of the kinetics of LC3B conversion by Western blot. Specifically, a time-dependent accumulation of LC3B-II was observed upon exposure of ES-1 cells to 100 μM EPZ-011989 (Figure 4D). A maximum peak of LC3B-II protein was appreciable at 48 h of drug exposure (Figure 4D), followed by an almost complete reduction of protein levels at 96 h, consistent with the presence of an active autophagic flux [38,39] (Figure 4D). On the other hand, exposure of ES-1 cells to a non-toxic concentration of Bafilomycin A1 (BafA1)—an inhibitor of the late steps of the autophagic process [38]—resulted in an impeded autophagic flux. Indeed, a time-dependent increase of LC3B-II protein amounts, with a maximum peak at 48 h of drug exposure followed by steady-state protein levels at later time points, was readily appreciable (Figure 4D). In addition, ES-1 cells exposed to the BafA1-EPZ-011989 combination showed the same kinetics of LC3B-II accumulation observed in BafA1-treated cells (Figure 4D), thus suggesting that the authophagy inhibitor efficiently counteracted the EPZ-011989-mediated autophagic flux. As expected, BafA1 exposure did not modify the EPZ-011989 effect on the methylation status of H3K27. Importantly, when ES-1 cells were concomitantly exposed to EPZ-011989 and BafA1, a significantly increased growth inhibition was observed compared with cells treated with single agents (Figure 4E). Similarly, the knock-down of the *ATG5* gene (Figure S2)—a member of the autophagy-specific *ATG* gene family, which is involved in the early steps of autophagosome formation [38]—was sufficient to revert the autophagic phenotype of EPZ-011989-treated cells, as revealed by the reduced LC3B conversion (Figure 4F). This evidence indicated that *ATG5* knock-down was able to counteract EPZ-011989-mediated induction of autophagy, resulting in a significant increase in ES-1 cells’ sensitivity to EPZ-011989 compared to ATG5-competent cells (Figure 4G).

Overall, these findings corroborate the initial hypothesis on the protective role of autophagy, which may become activated by ES cells in their attempt to cope with the growth inhibitory effect of the EZH2 inhibitor. Interestingly, treatment of ES-1 cells with equitoxic concentrations of gemcitabine, doxorubicin, and 4-hydroperoxycyclophosphamide (corresponding to IC80 values determined after a 72-h exposure to each drug) caused a negligible effect on LC3B-II accumulation compared to EPZ-011989, suggesting that the autophagic response was exclusively induced by EPZ-011989 in these cells (Figure 4H). Conversely, no sign of senescence was observed in ES-1 cells after 96 h of treatment with 100 µM EPZ-011989, as assessed by SA-β-Gal staining (Figure S3).

#### 2.2.5. *HMGA2* Is a Main Player in EPZ-011989-Induced Autophagy

**Figure 5:**
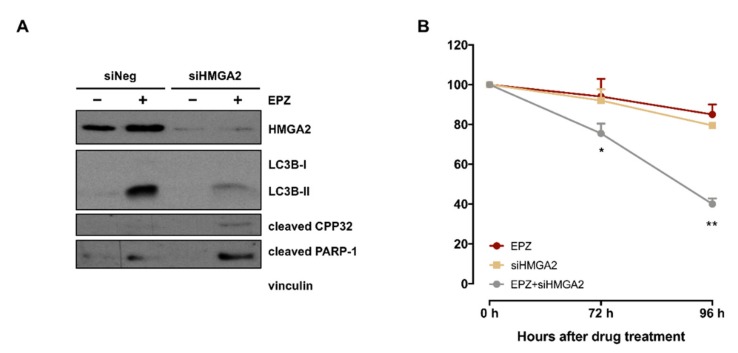
Effects of *HMGA2* silencing on EPZ-011989-induced autophagy in a PDX-derived ES-1 cell line. (**A**) Western blots showing the expression of HMGA2, LC3B, cleaved PARP-1 and cleaved caspase 3 (CPP32) in untreated and EPZ-011989 (100 µM for 96 h)-treated ES-1 cells after transfection with siHMGA2 or siNeg. Cropped images of selected proteins are shown. A representative Western blot of three independent experiments is shown. (**B**) Cell growth inhibition curves obtained after exposure to 100 µM EPZ-011989 of ES-1 cells transfected with siHGMA2 or siNeg. Data are reported as a percentage of EPZ-011989-treated cells (mean ± SD of three independent experiments) compared with untreated siNeg cells. * *p* < 0.05; ** *p* < 0.01.

The possible relevance of *HMGA2*, the fourth most up-regulated gene in tumors obtained from EPZ-011989-treated mice (Table 2), in the drug-induced autophagy activation was investigated by siRNA-mediated gene knock-down in ES-1 cells. Interestingly, the abrogation of HMGA2 protein expression resulted in a pronounced reduction of EPZ-011989-mediated LC3B conversion and was paralleled by an increased apoptotic response, as indicated by the higher abundance of cleaved forms of caspase 3 (CPP32) and poly (ADP-ribose) polymerase-1 (PARP-1) (Figure 5A). Consistently, *HMGA2* suppression also induced a significantly increase in the growth inhibitory effect of EPZ-011989 (Figure 5B), suggesting a functional involvement of the gene in drug-induced protective autophagy.

## 3. Discussion

This is the first ES PDX model reported so far. While the efficacy of EZH2 inhibitors has been described in ongoing clinical trials, we provide the first insight into the mechanism of EZH2 inhibition-induced tumor cell death and show evidence of a possible resistance mechanism in an INI-1 deficient P-ES PDX. In addition, our study shows marked antitumor activity of gemcitabine and of the doxorubicin–ifosfamide combination in the in vivo model. These results are consistent with clinical data [12] and show the ability of the P-ES PDX to reproduce the activity of chemotherapeutic agents clinically used for ES treatment.

PDXs are robust preclinical models which retain the main molecular and histologic features of clinical tumors, closely recapitulating the original heterogeneity. They are more reliable in predicting the response to therapeutic agents than cell line-derived xenografts. The availability of translatable preclinical models of very rare diseases, such as ES, could certainly accelerate drug development and prioritize suitable treatment strategies to be tested in clinical trials. As an example, we previously showed the consistency between preclinical data obtained on PDXs of a solitary fibrous tumor—another very rare STS subtype—and clinical results concerning the activity of several cytotoxic and anti-angiogenic agents [26,40,41], providing novel insight into the antitumor effect of different combinations that were instrumental to design currently ongoing clinical studies. Consistently, it is very interesting to note that the patient from whom the ES PDX was derived had a major response to chemotherapy with doxorubicin plus ifosfamide and to gemcitabine and, successively, a prolonged stable disease after treatment with an EZH2 inhibitor.

In the present study, consistent with ES dependence on EZH2 as a consequence of INI1 deficiency [16], we observed significant antitumor activity of EPZ-011989 in our PDX model, although with different kinetics of tumor growth inhibition compared to chemotherapeutics. Indeed, the compound initially caused a slight tumor growth delay, which was followed by a progressive reduction of tumor volume that started from the end of treatment. The EPZ-011989-induced antitumor effect was sustained at a biochemical level by a marked inhibition of H3K27 trimethylation and increased H3K27 acetylation, which was still appreciable when H3K27me3 expression was already partially recovered. Consistent with this epigenetic shift, RNAseq revealed a significantly increased transcriptional activity in tumors obtained from EPZ-011989-treated mice compared to those from untreated mice.

In spite of a significant tumor growth delay induced by EPZ-011989, tumor samples excised from mice at different intervals from the end of EPZ-011989 treatment were characterized by a high percentage of viable tumor cells comparable to that of untreated tumors, although with a lower proliferative potential, as indicated by a reduced Ki67 index. Based on such evidence, we hypothesized that ES cells could activate a cytoprotective mechanism to counteract the effects induced by EPZ-011989 treatment. This hypothesis was corroborated by transcriptome analysis results indicating a positive enrichment of genes involved in autophagy-related pathways in tumors obtained from EPZ-011989-treated mice. In addition, biochemical analysis carried out in treated samples showed an increased accumulation of the autophagy marker LC3B-II. The activation of autophagy as a cytoprotective mechanism against EPZ-011989 was functionally validated in the ES-1 cell line obtained from the PDX. Combined exposure of ES-1 cells to EPZ-011989 and BafA1, a late-stage autophagy inhibitor that prevents the fusion between autophagosomes and lysosomes [38], caused a significantly increased growth inhibition compared to that induced by EPZ-011989 alone. Similar results were obtained in ES-1 cells exposed to EPZ-011989 after silencing of the *ATG5* gene, an *ATG* family member which is involved in the early steps of autophagosome formation [38]. These findings are in line with previous evidence indicating that histone modification plays an important role in the regulation of autophagy and that EZH2 acts as a negative regulator of this process [37]. Although previous studies linked EZH2 to autophagy [42,43,44], this is the first evidence obtained in ES models.

In the search for main players in EPZ-011989-induced autophagy, we focused on *HMGA2*, based on the evidence that (i) it is the fourth most up-regulated gene by the drug, and (ii) the HMGA2 protein is a structural transcription factor, which is involved in gene transcription regulation, chromatin condensation and DNA damage repair [45]. *HMGA2* was also recently shown to play an important role in Chromium (VI)-induced autophagy [31]. *HMGA2* silencing in ES-1 cells almost completely abolished LC3B-II accumulation, induced apoptosis, and significantly increased the growth inhibitory effect of EPZ-011989, thus revealing a major role in sustaining EPZ-011989-induced autophagy.

As recently suggested in the literature, the antitumor activity of EZH2 inhibitors could rely in part on tumor-infiltrating Treg reprogramming and consequent enhancement of anti-cancer immunity [46]. Due to the use of immunodeficient mice to grow our PDX model, we are not able to reproduce such findings. Another limitation of our in vivo data is related to the intrinsic PDX model characteristics, which specifically recapitulate the features of INI1-deficient, *SMARCB1*-undeleted P-ES, highlighting the need to confirm our preclinical findings in a D-ES model.

## 4. Materials and Methods

### 4.1. Experimental Models

An INI1-deficient P-ES sample suitable for mouse implantation was obtained from the primary tumor of an adult patient surgically resected for a naïve ES arising from the forearm.

The clinical history of the patient, a 28-year-old male at the time of the initial diagnosis, was marked by multiple locoregional multifocal recurrences occurring after the resection of the primary tumor, which were treated with multimodal approaches including medical therapy. Path diagnosis at the time of disease onset was INI-deficient P-ES. Medical treatment included full-dose anthracycline (epirubicin) plus ifosfamide for 6 cycles with a RECIST partial response (PR) lasting 9 months, low dose weekly gemcitabine for 6 cycles with a RECIST PR followed by surgery, and an EZH2 inhibitor received within a clinical study with a stable disease lasting 8 months.

The use of patient material to generate the PDX model and all in vivo experiments were approved by the Ethics Committee for Animal Experimentation of Fondazione IRCCS Istituto Nazionale dei Tumori of Milan, Italy (INT code:16/17), according to institutional guidelines that are in compliance with national and international laws and policies. All experiments were carried out using female SCID mice that were 6–8 weeks old (Charles River, Calco, Italy). Mice were maintained in a pathogen-free facility where temperature and humidity were kept constant. Mice had free access to food and water. Experiments were authorized by the Italian Ministry of Health according to the national law (Project approval code: 234/2018-PR, approved on 27 March 2018) in compliance with international policies and guidelines.

#### 4.1.1. Development of PDX and PDX-Derived In Vitro Cell Line

A fresh tumor specimen was collected at the time of the surgical resection, aseptically dissected, cut into small fragments (3 × 3 × 3 mm) and grafted subcutaneously into the right flank of 6–8-week-old female SCID mice. Tumor growth was followed by biweekly measurement of tumor diameters with a Vernier calliper. Tumor volume (TV) was calculated according to the formula: TV (mm^3^) = d2 × D/2, where d and D are the shortest and the longest diameters, respectively. After the third passage in mice, and based on growth characteristics, the PDX was considered established.

To generate the corresponding cell line, the PDX was enzymatically digested with collagenase (200 U/mL) (Sigma, St. Louis, MO, USA) for 3 h at 37 °C. Collagenase was inactivated by two washes in fetal bovine serum (FBS). Cells were then resuspended in complete DMEM (Lonza, Verviers, Belgium). When cells reached 70–80% confluence, they were propagated under optimal culture conditions. The origins of both PDX and the cell line were authenticated through microsatellite analysis by the AmpFISTR Identifiler PCR amplification kit (Applied Biosystems, PN4322288, Foster City, CA, USA).

#### 4.1.2. Characterization of the PDX Model

Tumor tissue from sacrificed mice was formalin-fixed and paraffin-embedded. Four micrometer sections were colored with hematoxylin and eosin (H and E) for morphological evaluation and immunostained with INI1 (Ab INI1, Clone 25, #612110 Becton Dickinson, Lexington, KY, USA; dilution 1:200), H3 trimethyl K27 (Ab H3K27me3, Clone C36B11, #9733 Cell Signaling, Beverly, MA, USA; dilution 1:400) and Ki67 (Ab Ki67, Clone Mib1, #GA626 Dako, Santa Clara, CA, USA; dilution 1:400) for further characterization.

Immunohistochemical (IHC) analysis was performed at room temperature on the Dako Autostainer Link 48 AS480 (Agilent, Santa Clara, CA, USA). Once the IHC procedure was initiated, the slides were rinsed with buffer immediately before and after each of the following steps: (1) Incubation with Envision Peroxidase (Dako) for 5 min to quench endogenous peroxidase; (2) incubation with primary antibody for 30 min; (3) incubation with Envision FLEX Linker for 15 min; (4) incubation with Envision FLEX horseradish peroxidase for 20 min; and (5) detection with diaminobenzidine (Dako) for 10 min each. Upon completion of the staining procedure, slides were counterstained offline with hematoxylin (Dako) for 2 min, rinsed, and coverslipped.

FISH assay was carried out to assess *SMARCB1* gene deletion in the clinical tumor of the patient and in the PDX model using a specific SPEC *SMARCB1*/22q12 Dual colour Probe (ZytoLight/ZytoVision, Bremerhaven, Germany), according to manufacturer’s instructions. The probe was a mixture of a green fluorochrome labelled to hybridize *SMARCB1* gene on 22q11.23 chromosome and an orange fluorochrome labelled to hybridize the *KREMEN1* gene localized on 22q12. The latter is useful for detecting chromosome 22 copy number deletion.

Ki67 labeling index (number of Ki67-positive nuclei/overall number of nuclei ×100) was quantified using ImageJ 1.47q software (https://imagej.nih.gov).

### 4.2. Pharmacology Studies

#### 4.2.1. In Vivo Evaluation of Drug Activity

Mice were randomized to receive different drugs when xenografts were approximately 150 mm^3^. Eight mice for the experimental group were used. The treatment dose and schedule for each drug were selected from literature (Table 1). After dilution in sterile water (doxorubicin (Adriblastina, Pfizer Italia) and ifosfamide (Holoxan (Baxter, Deerfield, IL, USA)), saline solution (gemcitabine (Gemzar, Eli Lilly Italia)) or 0.5% carboxymethylcellulose and 0.1% Tween 80 (EPZ-011989, kindly provided by Epizyme (Cambridge, MA, USA)), drugs were administered as follows: Doxorubicin was delivered intravenously (i.v.) every 7 days, three times (q7d × 3); ifosfamide was administered intraperitoneally (i.p.) 3 days/week, 2 times, after a 2-week rest (qd × 3 q 2w). The same doses and schedules were used when the two drugs were administered as single agents and in combination. Gemcitabine was administered i.v. every 4 days, four times (q4d × 4); EPZ-011989 was delivered orally (p.o.) twice a day for 28 consecutive days (2qd × 28) (Table 1).

Drug treatment activity was assessed in terms of TV inhibition percentage (TVI%) in treated versus control mice, expressed as TVI% = 100−[(mean TV treated/mean TV control) × 100]. Treatment toxicity was determined in terms of body weight loss and lethal toxicity.

#### 4.2.2. RNA Sequencing and Bioinformatics Analysis

Whole-transcriptome sequencing (WTS) was performed using the Illumina Nextseq500 platform (Illumina, San Diego, CA, USA). Total RNA was extracted from fresh–frozen tumors grown in two control (untreated) and two EPZ-011989-trated mice (at the end of treatment) with an RNeasy mini kit (Qiagen, Hilden, Germany). cDNA libraries were synthesized from 500 ng total RNA with TruSeq Stranded mRNA kit (Illumina, San Diego, CA, USA), following manufacturers’ instructions. Libraries were quantified by Quant-It picogreen assay (Thermo Fisher Scientific Inc, Waltham, MA, USA) and sized with the High Sensitivity kit on the 2100 Bioanalyzer (Agilent Technologies, Santa Clara, CA, USA). Sequencing was performed at 2 × 75bp using Illumina Sequencing by synthesis (SBS) technology.

Bioinformatics analysis was performed on local server CentOS5 Linux by applying a customized bioinformatics pipeline. After the bcl to fastq conversion, reads were cleaned and trimmed with AdapterRemoval (http://adapterremoval.readthedocs.io). Sequences were then aligned against human reference genome hg19 with STAR (https://github.com/alexdobin/STAR), adopting a specific strategy to eliminate the murine RNA contamination [47]. Differential expression was estimated both in terms of fold change (FC) and *t*-value, using the edgeR Bioconductor package [48]. Significance was provided in terms of false discovery rate (FDR) to take into account the adjustment for multiple hypotheses testing, using a threshold of 0.05. All these analyses were conducted in the R environment.

A *t*-value pre-ranked Gene Set Enrichment Analysis (GSEA) [49] was carried out using C2 gene sets v6.2 of the Molecular Signature database (MSigDB) or custom signatures. Custom-defined gene sets were obtained by selecting a list of genes involved in autophagy process according to Orlotti et al. [39], by interrogating the MSigDB with “autophagy” as query, and by selecting all gene sets of C2 collection containing the *EZH2* gene in their list. An FDR q-value threshold of 0.05 was used to define a significant enrichment.

The RNA-Seq dataset generated in the study was deposited in the GEO database (GSE126495).

#### 4.2.3. Cell-Based Studies

The growth inhibitory effect of 100 µM EPZ-011989, alone or in association with 30 nM Bafilomycin A1 (BafA1, Sigma, St. Louis, MO, USA), doxorubicin, 4-hydroperoxycyclophosphamide (Cayman Chemicals, Michigan, Ann Arbor, MI, USA), and gemcitabine were evaluated at 96 h of drug exposure by cell counting using a particle counter (Beckman Coulter, Luton, UK). The activities of drugs were expressed as percentages of adherent treated cells compared to untreated control cells. The highest concentration of DMSO that we used was 0.5%.

Small interfering RNAs (siRNAs) were used to knock-down *ATG5* (siATG5 [37], Eurofins Genomics) and *HMGA2* genes (siHMGA2, ON-TARGET plus SMART pool, Dharmacon, Cornaredo, Italy). A control siRNA (siNEG), with no homology to any known human mRNA, was used as control. Cells were transfected for 24 h with 20 nM siRNAs using Lipofectamine RNAiMAX Transfection Reagent (Thermo Fisher Scientific Inc, Waltham, MA, USA), according to the manufacturer’s protocol. Forty-eight hours after transfection, cells were treated with 100 µM EPZ-011989. At different time points after drug exposure, cells were counted to assess the effect on cell growth and collected for protein extraction and Western blot analysis.

For autophagy assessment, 96 h after 100 µM EPZ-011989 treatment, cells were incubated with 250 nM Lysotraker Red (Thermo Fisher Scientific Inc, Waltham, MA, USA) for 1 h and lysosomal staining was examined under a fluorescence microscope (Nikon Eclipse E600). In parallel, cells were fixed in 2% paraformaldehyde, permeabilized with 0.5% Triton X-100, and probed with an anti-LC3B polyclonal antibody (Cell Signaling). Cells were then labeled with secondary Alexa Flour 488 antibody (Thermo Fisher Scientific Inc, Waltham, MA, USA). Nuclei were counterstained with Hoechst 33,342 (Sigma, St. Louis, MO, USA) and examined under a fluorescence microscope (Nikon, Minato, Tokyo, Japan).

For senescence assessment, cells exposed to 100 µM EPZ-011989 for 96 h were fixed in 2% formaldehyde/0.2% glutaraldehyde, stained with fresh senescence-associated β-Galattosidase (SA-β-Gal) solution (Sigma, St. Louis, MO, USA) and incubated at 37 °C (no CO_2_). After 18 h, cell positivity to SA-β-Gal was examined under a microscope (Nikon, Minato, Tokyo, Japan).

#### 4.2.4. Western Blot Analysis

Lysates were obtained from frozen PDX tumors collected immediately and after 15 days from the end of treatment with EPZ-011989 as well as from the PDX-derived cell line after different intervals of exposure to EPZ-011989, alone or in association with Baf1A, or following transfection with siATG5 or siHMGA2. Lysates from ES-1 cells exposed to doxorubicin, 4-hydroperoxycyclophosphamide, and gemcitabine were also collected. Proteins were separated by SDS-PAGE, transferred onto nitrocellulose membranes and incubated with primary monoclonal antibodies anti-EZH2 (#5246, Cell Signaling), anti-H3K27me3 (#9733, Cell Signaling), anti-H3 acetyl K27 (H3K27ac, #8173, Cell Signaling), anti-EGR1 (#4153, Cell Signaling), anti-LC3B (#2775 Cell Signaling), anti-HMGA2 (#5269, Cell Signaling), anti-cleaved CCP32 (#9661, Cell Signaling), anti-cleaved PARP (#9541, Cell Signaling), anti-ATG5 (A0856, Sigma, St. Louis, MO, USA), anti-β-tubulin (T5201, Sigma, St. Louis, MO, USA), anti-β-actin (A2066, Sigma, St. Louis, MO, USA), and anti-vinculin (V9131, Sigma, St. Louis, MO, USA).

### 4.3. Statistical Analysis

Analyses by two-sided Student’s *t*-test were performed using the GraphPad Prism software, version 4.0 (GraphPad Prism Inc., San Diego, CA, USA). A *p* value of ≤0.05 was considered statistically significant.

## 5. Conclusions

Overall, results from this study confirm preclinically the activity of the doxorubicin–ifosfamide combination and gemcitabine in INI1-deficient P-ES. In addition, preclinical results also show that the inhibition of EZH2 with EPZ-011989 has antitumor activity comparable to that observed with chemotherapeutics and suggest the induction of autophagy as a possible mechanism of survival in residual tumor cells following EPZ-011989 treatment, supporting future studies on optimal combinations.

## Figures and Tables

**Table 1:** Pharmacological treatment and tumor responses of ES-1 PDX to different agents.

Treatment	Dose (mg/kg)	Schedule	Route	Max TVI% (Day) ^a^	Ref. ^b^
Doxorubicin	4.0	*q7d × 3*	*i.v.*	62 (43) *	[26]
Ifosfamide	90	*qd × 3 q 2w*	*i.p.*	75 (43) *	[26]
Ifosfamide	90	*qd × 3 q 2w*	*i.p.*	94 (43) **	[26]
Doxorubicin	4.0	*q7d × 3*	*i.v.*		[26]
Gemcitabine	100	*q4d × 4*	*i.v.*	98 (46) **	[27]
EPZ-011989	500	*2qd (b.i.d.) × 28*	*o.s.*	89 (50) **	[25]

**^a^** Maximum tumor volume inhibition (TVI) % in treated versus control mice. In parentheses, the day on which it was assessed. ^b^ References reporting treatment dose and schedule for each drug. * *p* < 0.01, ** *p* < 0.001 versus control tumors.

**Table 2:** The top 15 up-regulated genes in EPZ-011989-treated tumor samples according to the *t*-value statistics; eight of them have an association with cell survival pathways, as described in the quoted references.

Gene	*t*-Value	Ref.
*IGFBP4*	39.40	[29]
*DCN*	31.83	[30]
*CALD1*	29.15	
*HMGA2*	29.10	[31]
*FAM84B*	28.83	
*SLIT3*	27.53	
*MMP16*	27.35	
*FOLH1*	27.27	
*BICC1*	26.90	
*COL21A1*	26.83	
*EFEMP1*	26.45	[34]
*INPP4B*	26.24	[35]
*INSIG2*	25.27	[36]
*SPARC*	25.24	[32]
*ALDH1A1*	24.55	[33]

## Supplementary Materials

The following are available online at https://www.mdpi.com/2072-6694/11/7/1015/s1, Figure S1: (*Top*) Customly defined or MSigDB signatures related to “autophagy” showed a positive enrichment trend in genes up-regulated upon EZH2 inhibition by EPZ-011989. (*Bottom*) Table describing statistical features such as the size of the gene set, the Normalized Enriched Score (NES) and the FDR q-value; a threshold of 0.05 was used to assess the significance of the enrichment, Figure S2: ES-1 cells were transiently transfected with control or ATG5-directed siRNAs, Figure S3: S-β-gal staining of untreated and EPZ-011989 (100 µM for 96 h)-treated ES-1 cells, Table S1: Table showing GSEA carried out on genes up- or down-regulated upon EZH2 inhibition by EPZ-011989, Table S2: Table showing the custom-defined signature of 84 genes associated to autophagy, Table S3: Table reporting RNA-seq results.
