# Supplementary material for: Comparative Assessment of Antitumor Effects and Autophagy Induction as a Resistance Mechanism by Cytotoxics and EZH2 Inhibition in INI1-Negative Epithelioid Sarcoma Patient-Derived Xenograft
Source: Cancers (Basel). 2019 Jul 19;11(7):1015. doi: 10.3390/cancers11071015 (PMC6678245; doi:10.3390/cancers11071015)
Supplement: Supplementary file 1 [file cancers-11-01015-s001.zip › Supplementary figures.pdf]

Supplementary Materials

Comparative Assessment of Antitumor Effects and Autophagy Induction as a Resistance Mechanism by Cytotoxics and EZH2 Inhibition in INI1-Negative Epithelioid Sarcoma Patient-Derived Xenograft

Silvia Stacchiotti, Valentina Zuco, Monica Tortoreto, Denis Cominetti, Anna Maria Frezza, Stefano Percio, Valentina Indio, Marta Barisella, Valentina Monti, Silvia Brich, Annalisa Astolfi, Chiara Colombo, Sandro Pasquali, Marco Folini, Mrinal M. Gounder, Maria A. Pantaleo, Paola Collini, Angelo Paolo Dei Tos, Paolo Giovanni Casali, Alessandro Gronchi and Nadia Zaffaroni

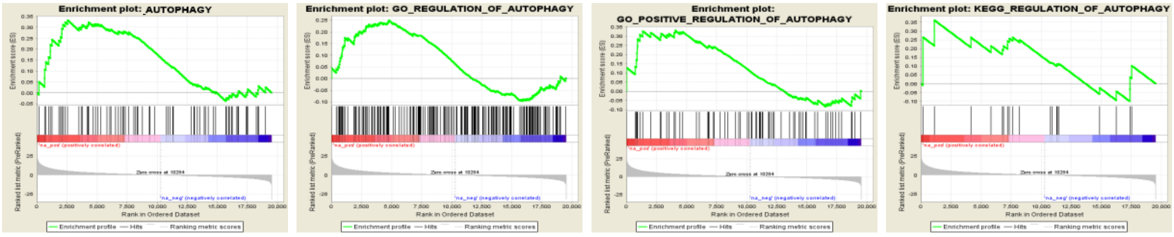

Gene Set	Description	SIZE	NES	FDR q-val
AUTOPHAGY	Regulation of autophagy (home-made list)	84	1.11	0.29
GO_REGULATION_OF_AUTOPHAGY	Any process that modulates the frequency, rate or extent of autophagy. Autophagy is the process in which cells digest parts of their own cytoplasm.	234	0.91	0.64
GO_POSITIVE_REGULATION_OF_AUTOPHAGY	Any process that activates, maintains or increases the rate of autophagy. Autophagy is the process in which cells digest parts of their own cytoplasm.	74	1.09	0.68
KEGG_REGULATION_OF_AUTOPHAGY	Regulation of autophagy	21	0.95	0.69

Figure S1. *(Top)* Customly defined or MSigDB signatures related to "autophagy" showed a positive enrichment trend in genes up-regulated upon EZH2 inhibition by EPZ-011989. *(Bottom)* Table describing statistical features such as the size of the gene set, the Normalized Enriched Score (NES) and the FDR *q*-value; a threshold of 0.05 was used to assess the significance of the enrichment.

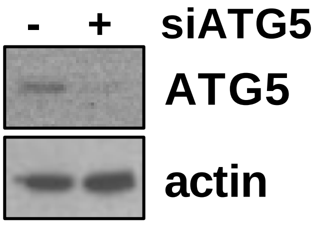

Figure S2. ES-1 cells were transiently transfected with control or ATG5-directed siRNAs. ATG5 levels were evaluated by Western blots after 72 h from transfection. A representative western blots of ATG5 and actin is reported.

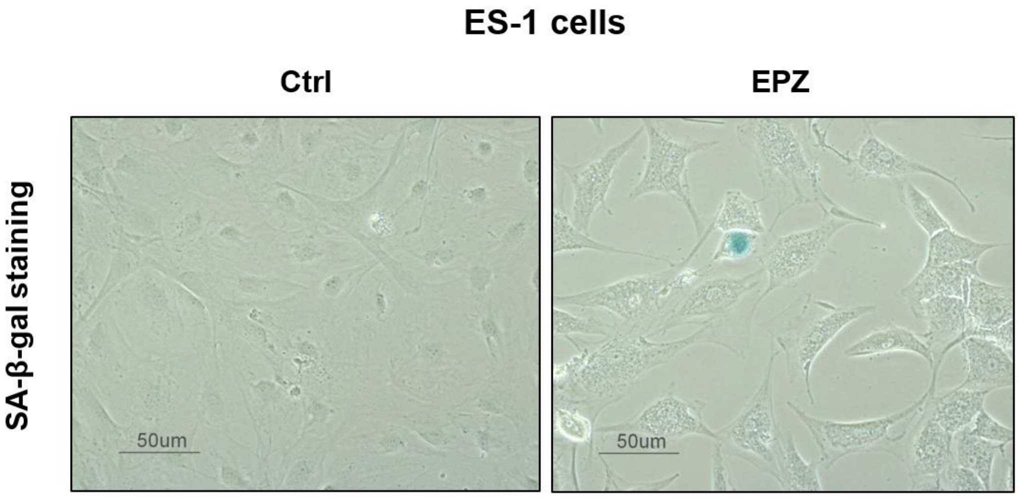

Figure S3. S-β-gal staining of untreated and EPZ-011989 (100 μM for 96 h)-treated ES-1 cells. Scale bar, 50 μm. One representative experiment is shown.

Whole blots showing all the bands of Western blotting presented in Figures 2,4,5 in the main text.

Figure 2B

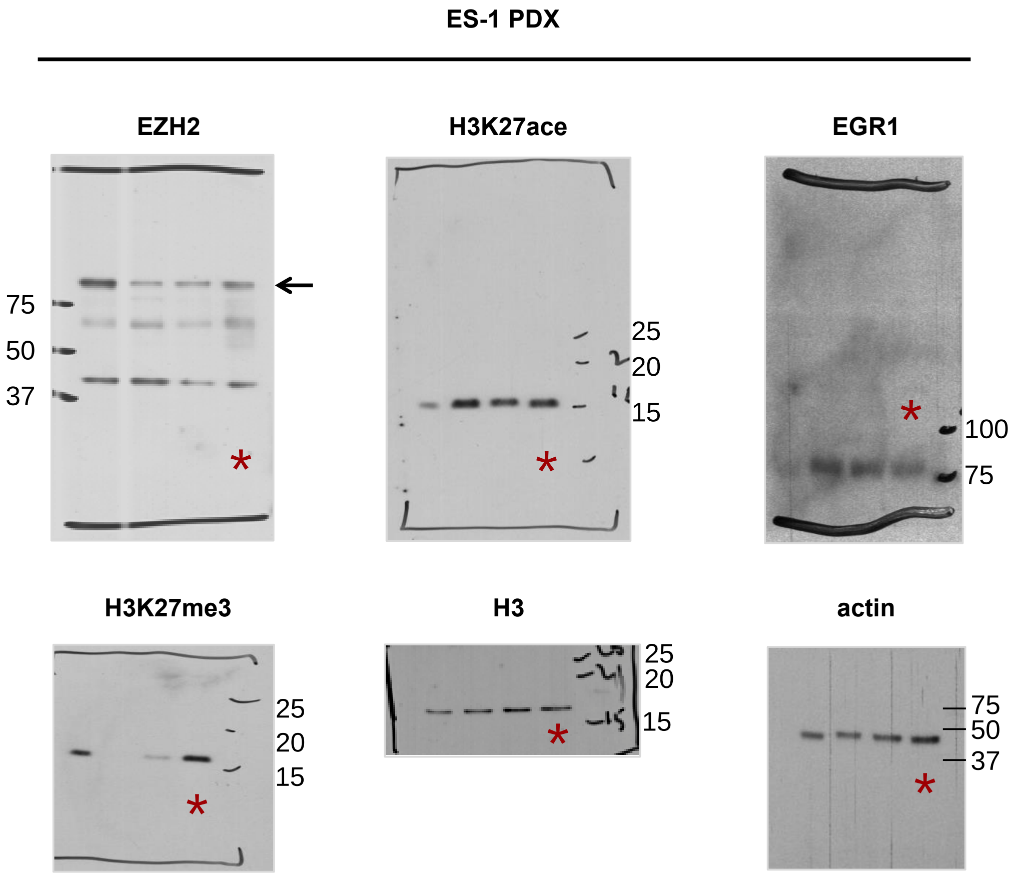

Figure 2B

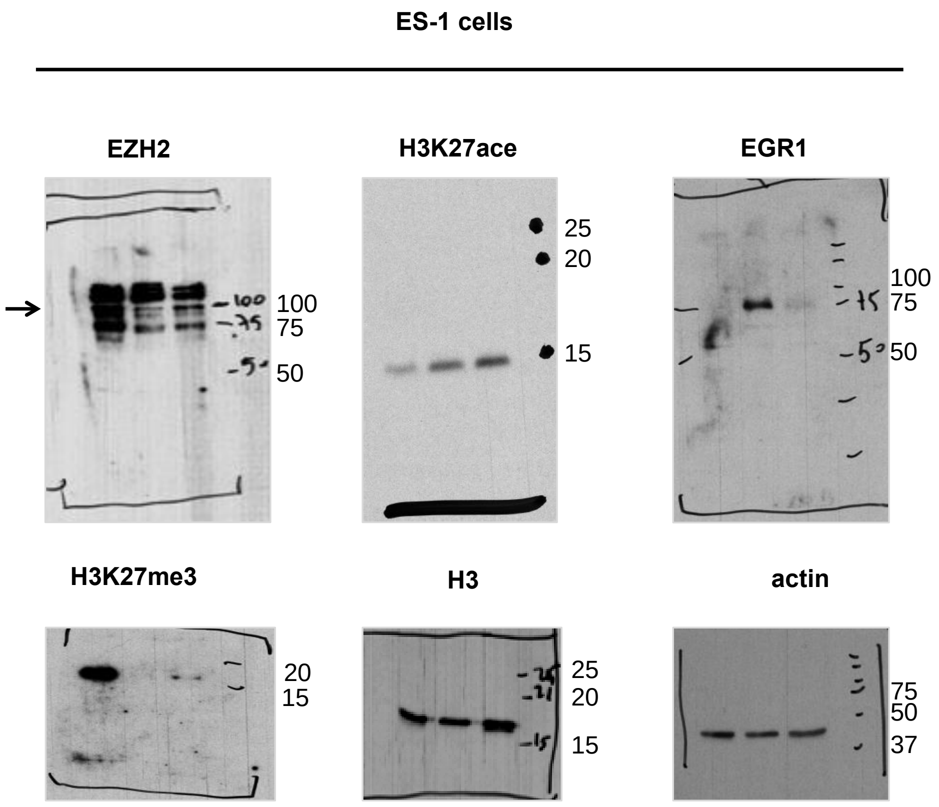
ES-1 cells
EZH2
100
75
50
H3K27ace
25
20
15
EGR1
100
75
50
H3K27me3
20
15
H3
25
20
15
actin
75
50
37

Figure 4 A,C

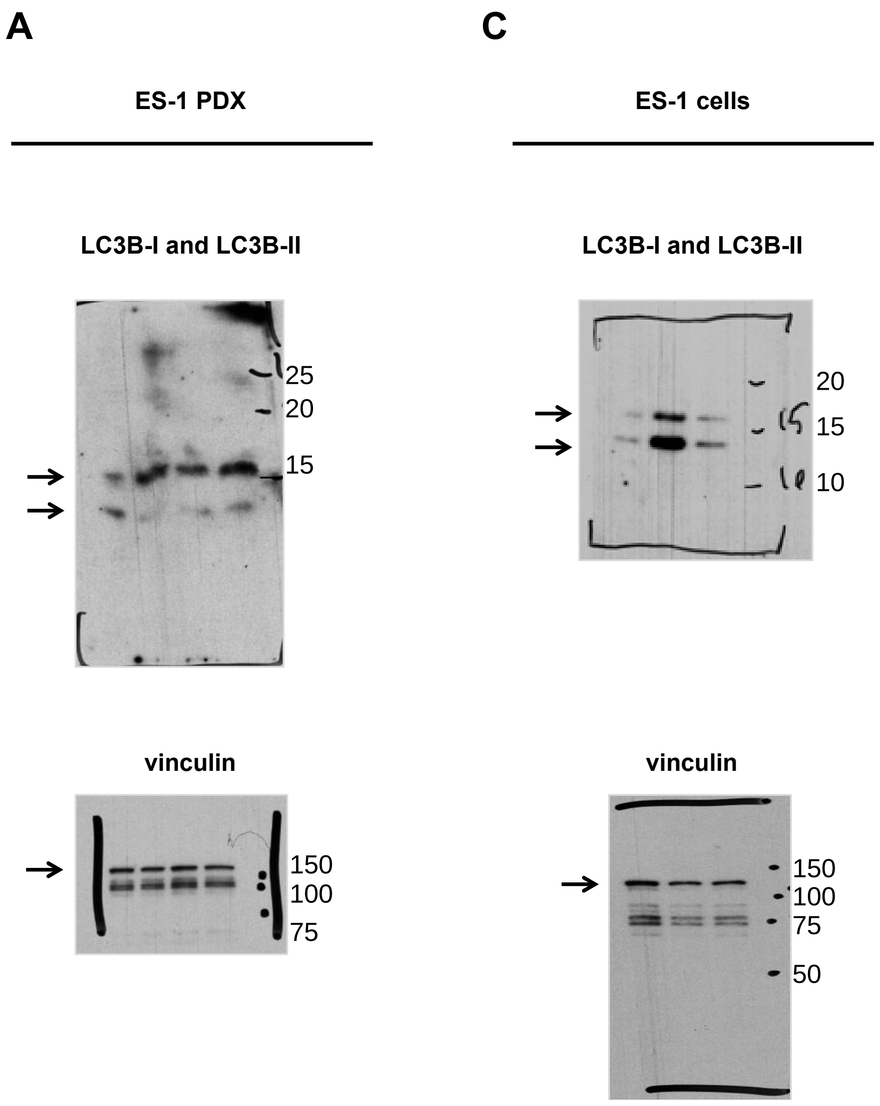
A
ES-1 PDX
LC3B-I and LC3B-II
25
20
15
vinculin
150
100
75
C
ES-1 cells
LC3B-I and LC3B-II
20
15
10
vinculin
150
100
75
50

Figure 4 D

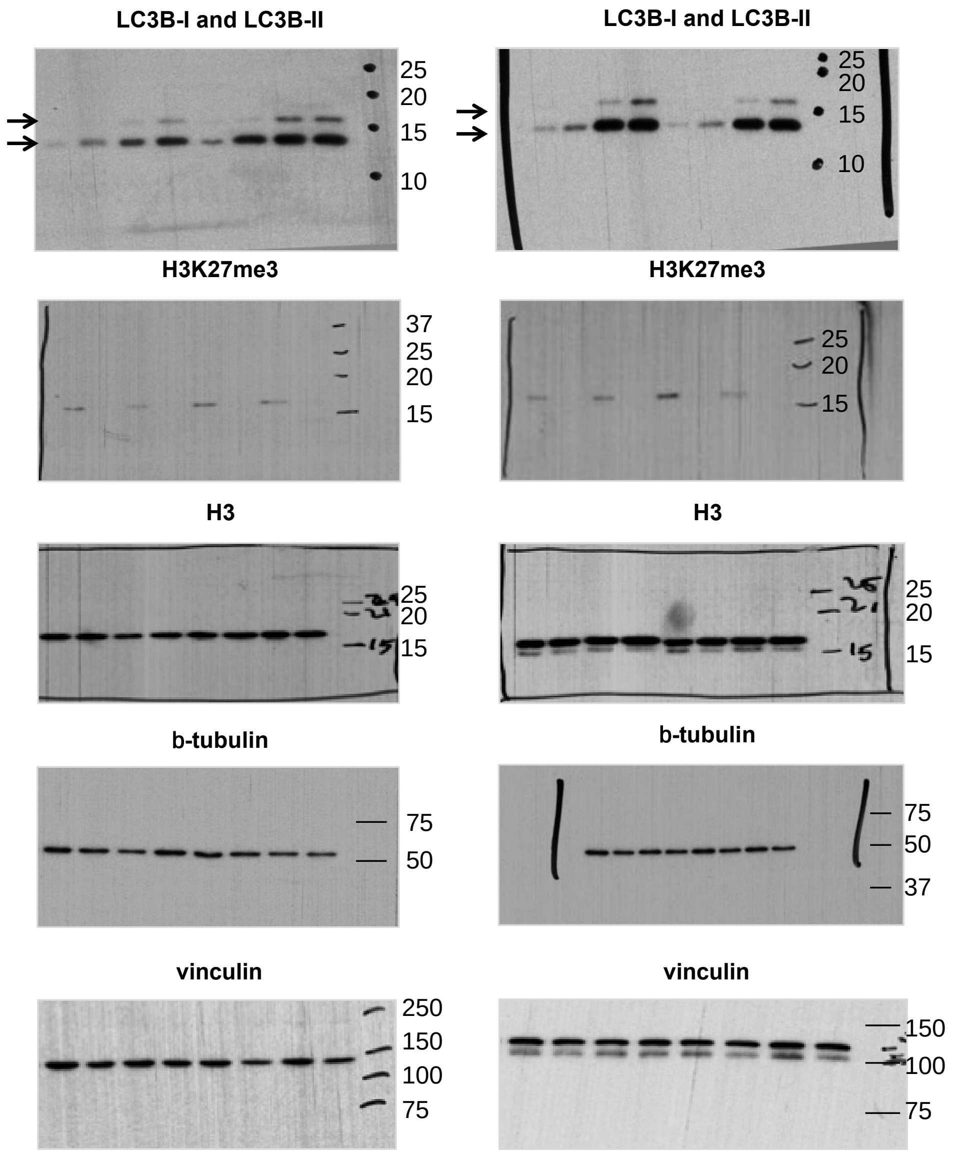
LC3B-I and LC3B-II
25
20
15
10
LC3B-I and LC3B-II
25
20
15
10
H3K27me3
37
25
20
15
H3K27me3
25
20
15
H3
25
20
15
H3
25
20
15
b-tubulin
75
50
b-tubulin
75
50
37
vinculin
250
150
100
75
vinculin
150
100
75

Figure 4F

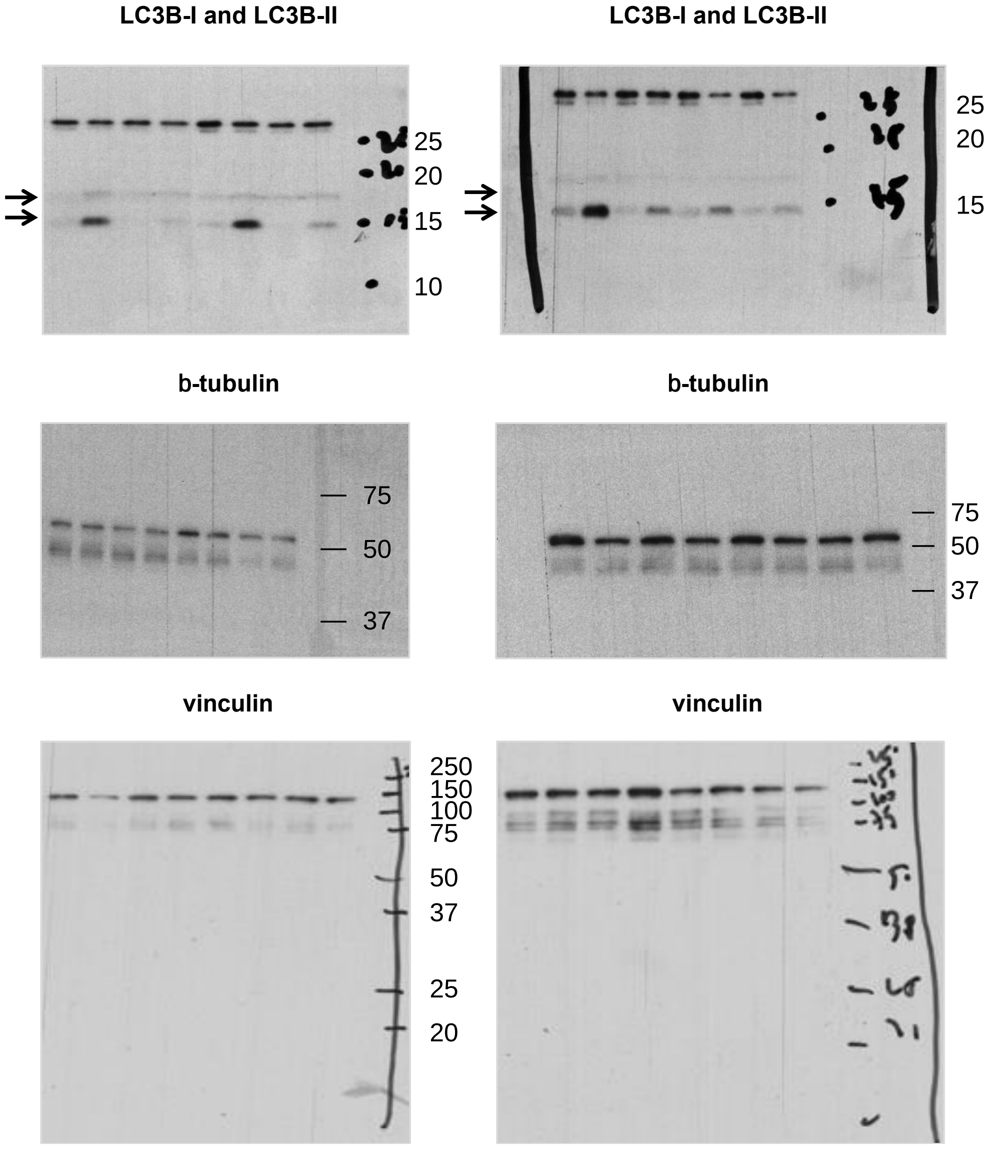
LC3B-I and LC3B-II
25
20
15
10
LC3B-I and LC3B-II
25
20
15
b-tubulin
75
50
37
b-tubulin
75
50
37
vinculin
250
150
100
75
50
37
25
20
vinculin

Figure 4H

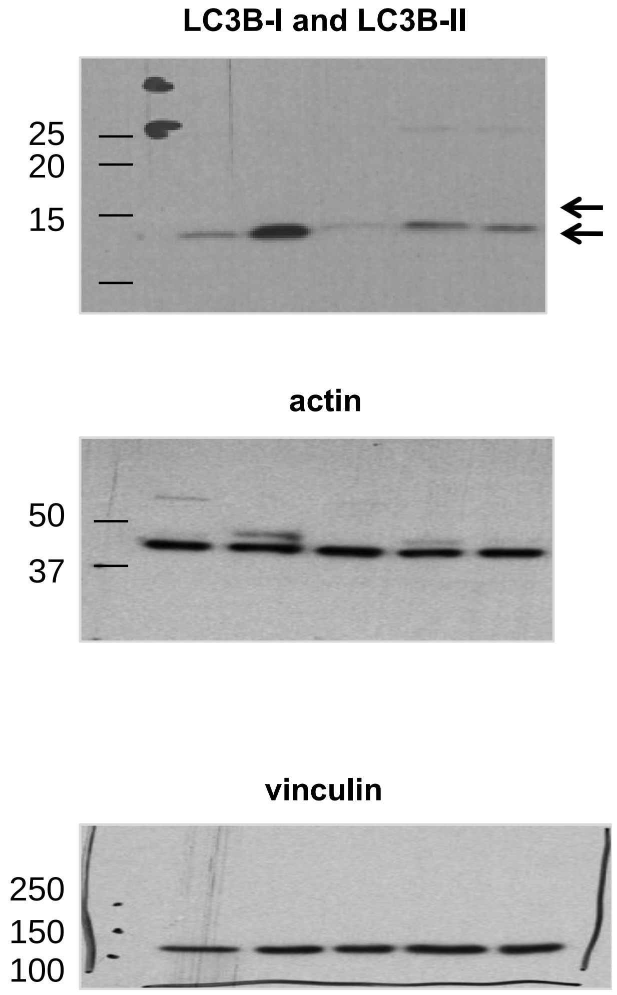

Figure 5A

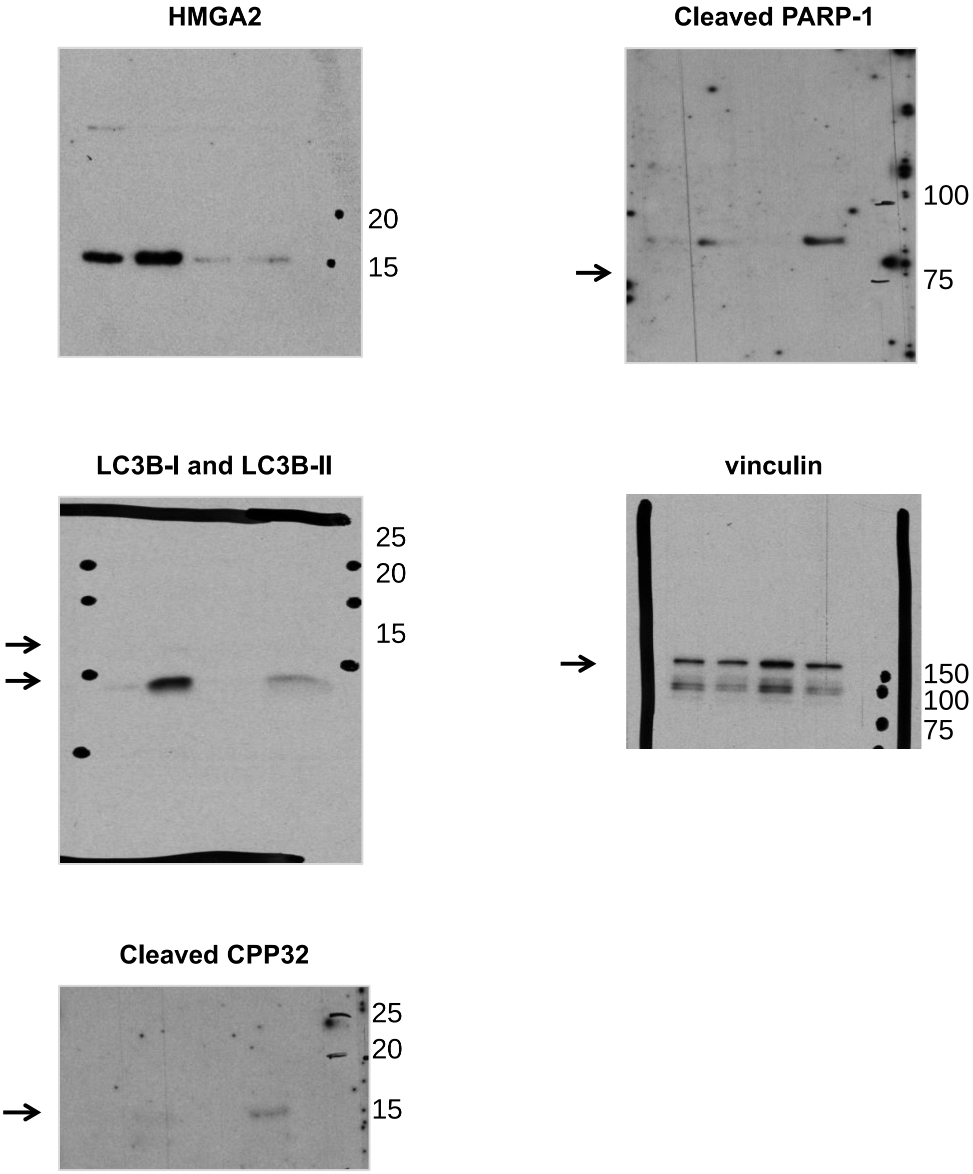
HMGA2
20
15
Cleaved PARP-1
100
75
LC3B-I and LC3B-II
25
20
15
vinculin
150
100
75
Cleaved CPP32
25
20
15

Band intensity quantification. Band intensities, quantified using ImageJ, were normalized to protein loading control. Actin (Figure 2) or Vinculin (Figures 4 and 5) was used as loading control.

Figure 2B

	ES-1 PDX		
days	-	0	15
EZH2	3.05	0.67	0.56
H3K27me3	2.13	0.02	0.59
H3K27 ac	0.57	2.67	1.29
H3	0.53	0.87	0.76
EGR1	0.38	2.88	1.39
H3K27me3/H3	4.04	0.02	0.78
H3K27ac/H3	1.08	3.06	1.70

	ES-1 cells		
EPZ (mM)	-	100	10
EZH2	2.67	0.63	2.14
H3K27me3	3.79	0.08	0.34
H3K27 ac	0.37	1.05	0.98
H3	4.81	3.68	5.24
EGR1	0.02	3.47	0.02
H3K27me3/H3	0.79	0.02	0.06
H3K27ac/H3	0.08	0.29	0.19

Figure 4A

	ES-1 PDX			
days	-	0	15	32
LC3B-I	0.64	1.27	0.96	1.30
LC3B-II	0.05	0.15	0.51	0.81
LC3B-II/LC3B-I	0.08	0.12	0.53	0.62

Figure 4C

	ES-1 ES		
EPZ (mM)	-	100	10
LC3B-I	0.05	1.05	0.17
LC3B-II	0.07	1.81	0.26
LC3B-II/LC3B-I	1.40	1.73	1.57

Figure 4D

	24h				48h				72h				96h			
EPZ	Neg	Pos	Neg	Pos	Neg	Pos	Neg	Pos	Neg	Pos	Neg	Pos	Neg	Pos	Neg	Pos
BafA1	Neg	Neg	Pos	Pos	Neg	Neg	Pos	Pos	Neg	Neg	Pos	Pos	Neg	Neg	Pos	Pos
LC3B-I	0.01	0.03	0.02	0.20	0.03	0.08	0.44	0.71	0.03	0.01	2.06	0.62	0.03	0.01	0.11	0.54
LC3B-II	0.10	0.50	0.72	1.21	0.25	2.02	1.40	1.85	0.12	0.36	1.41	1.35	0.07	0.29	1.28	1.71
H3K27me3	0.14	0.03	0.10	0.02	0.21	0.03	0.18	0.03	0.10	0.03	0.16	0.02	0.41	0.02	0.13	0.02
H3	0.53	1.04	0.54	0.58	0.53	1.02	0.56	0.87	0.45	0.64	0.71	0.81	0.68	0.58	0.55	0.94
LC3B-II/LC3B-I	7.55	19.63	32.27	5.91	9.79	26.35	3.20	2.58	4.56	32.52	0.69	2.18	2.51	35.72	11.19	3.15
H3met/H3	0.26	0.03	0.18	0.04	0.40	0.03	0.32	0.03	0.23	0.04	0.23	0.02	0.61	0.04	0.23	0.02

Figure 4F

	24h				48h				72h				96h			
EPZ	Neg	Pos	Neg	Pos	Neg	Pos	Neg	Pos	Neg	Pos	Neg	Pos	Neg	Pos	Neg	Pos
siATG5	Neg	Neg	Pos	Pos	Neg	Neg	Pos	Pos	Neg	Neg	Pos	Pos	Neg	Neg	Pos	Pos
LC3B-I	0.49	2.68	0.49	0.61	0.44	1.00	0.51	0.90	0.35	0.35	0.29	0.16	0.06	0.20	0.18	0.29
LC3B-II	0.06	2.97	0.03	0.10	0.05	1.40	0.02	0.31	0.26	1.31	0.08	0.44	0.12	0.67	0.13	0.32
LC3B-II/LC3B-I	0.12	1.11	0.07	0.17	0.12	1.40	0.05	0.34	0.73	3.73	0.28	2.73	1.96	3.35	0.71	1.13

Figure 4H

EPZ	-	+	-	-	-
GEM	-	-	+	-	-
DX	-	-	-	+	-
4-HCy	-	-	-	-	+
LC3B-I	0.0033	0.0041	0.0155	0.1493	0.0954
LC3B-II	0.09	0.41	0.06	0.18	0.11
LC3B-II/LC3B-I	29.04	99.49	3.61	1.18	1.18

Figure 5A

	siNeg		siHMGA2	
EPZ	-	+	-	+
HMGA2	0.81	1.50	0.07	0.10
Cleaved CPP32	0.10	0.26	0.05	0.65
Cleaved PARP-1	0.22	0.50	0.07	1.26
LC3B-I	1.00	1.21	0.81	0.83
LC3B-II	0.12	2.34	0.06	0.69
LC3B-II/LC3B-I	0.12	1.94	0.08	0.83

© 2019 by the authors. Licensee MDPI, Basel, Switzerland. This article is an open access article distributed under the terms and conditions of the Creative Commons Attribution (CC BY) license (http://creativecommons.org/licenses/by/4.0/).
